# Circulating Amino Acid Network Remodeling Reveals Systemic Metabolic Reprogramming Predictive of Colorectal Cancer Recurrence and Metastasis

**DOI:** 10.1002/advs.76044

**Published:** 2026-06-09

**Authors:** Ji‐Yeon Lee, Jumi Kim, Taehan Yoon, So Hyun Kwon, Su Chan Park, Dohyun Chun, Dohyeong Kim, Eun Jung Park, Hyunwoo Kim, Ji Min Lee

**Affiliations:** ^1^ Graduate School of Medical Science and Engineering Korea Advanced Institute of Science and Technology (KAIST) Daejeon Republic of Korea; ^2^ Department of Chemistry Korea Advanced Institute of Science and Technology (KAIST) Daejeon Republic of Korea; ^3^ Division of Colon and Rectal Surgery Department of Surgery Gangnam Severance Hospital Yonsei University College of Medicine Seoul Republic of Korea; ^4^ Division of Colon and Rectal Surgery Department of Surgery Asan Medical Center University of Ulsan College of Medicine Seoul Republic of Korea

**Keywords:** ^19^F NMR, circulating amino acids, colorectal cancer, metabolic alterations, prediction of progression risk

## Abstract

Characterizing the dynamics of systemic metabolic pathways during cancer progression could enable the development of prognostic tools and therapeutic strategies. However, achieving this goal requires analytical platforms optimized for liquid biospecimens together with interpretive frameworks capable of identifying robust serum‐based biomarkers. Here, we establish a network‐based metabolic profiling framework using ^1^ ^9^F NMR–based serum amino acid analysis to characterize systemic metabolic remodeling during colorectal cancer (CRC) progression. Using an analytical protocol optimized for clinical serum samples, we quantified circulating amino acids from 152 CRC patients and implemented a ratio‐based normalization strategy to mitigate cohort variability in concentration‐based approaches. We systematically evaluated both individual amino acid changes and correlation structures across tumor stages. Advanced‐stage CRC exhibited a distinct metabolic shift characterized by decreased valine and increased glycine levels. Correlation network analysis further revealed stage‐dependent remodeling of circulating amino acid interactions, leading to the emergence of a glycine‐centered metabolic architecture. Importantly, machine‐learning models integrating individual amino acid levels with network‐derived features significantly improved the prediction of recurrence or metastasis compared with models using either feature type alone and outperformed conventional biomarker carcinoembryonic antigen (AUROC = 0.806). These findings highlight the remodeling of circulating amino acid network as a promising strategy for prognostic stratification and postoperative monitoring in CRC.

## Graphical abstract

Blood‐based amino acid patterns measured by ^19^F NMR reveal hidden metabolic changes in colorectal cancer. By analyzing how these amino acids interact as a network, machine learning models identify patients at higher risk of recurrence and metastasis. This approach provides a minimally invasive strategy for clinical monitoring of recurrence and metastasis.

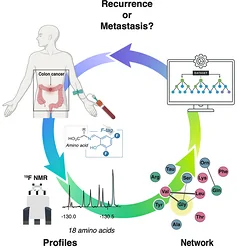

## 1 Introduction

Metabolic reprogramming is a defining feature of cancer, enabling tumor cells to sustain rapid proliferation despite restricted nutrient availability in the surrounding environment [1, 2, 3, 4]. Colorectal cancer (CRC), for example, exhibits metabolic profiles that differ markedly from those of normal tissues, reflecting adaptations to elevated oxidative stress and energetic demands [5, 6, 7, 8, 9]. A prominent aspect of this metabolic reprogramming is an increased dependency on exogenous amino acids, which support cancer cell bioenergetics, redox homeostasis, and biosynthetic processes [10, 11, 12]. In addition to reshaping the metabolic composition of the local microenvironment, tumors can also exert systemic metabolic effects that extend throughout the host [13, 14, 15]. These systemic alterations include fluctuations in circulating metabolites such as serum amino acids, which represent both substrates and products of cancer‐associated metabolic activity. Consequently, serum amino acid profiling may provide a minimally invasive window into the evolving metabolic states that accompany cancer progression and tumor–host metabolic interactions.

Previous studies have employed analytical platforms including gas chromatography–mass spectrometry (GC–MS), liquid chromatography–mass spectrometry (LC–MS), and proton nuclear magnetic resonance (^1^H NMR) spectroscopy to characterize serum metabolites associated with CRC, primarily aiming to identify diagnostic biomarkers [16, 17, 18, 19, 20, 21, 22, 23, 24, 25, 26, 27, 28]. Although these efforts have revealed differential metabolite signatures between CRC patients and healthy individuals, most studies have focused on individual metabolites or threshold‐based biomarker definitions. Such approaches overlook the intrinsic interdependence among metabolites within metabolic networks; therefore, they capture only a limited view of cancer‐associated metabolic states. Moreover, absolute metabolite concentrations are susceptible to physiological variability and technical inconsistencies across cohorts, potentially obscuring disease‐specific metabolic alterations. These limitations highlight the need for analytical frameworks capable of capturing system‐level metabolic relationships rather than relying solely on individual metabolite abundance. Approaches that evaluate changes in amino acid networks may therefore provide a more robust representation of systemic remodeling and stage‐dependent shifts in metabolic dependencies during cancer progression.

From a clinical perspective, accurate staging and prognostic assessment are essential for guiding treatment strategies and improving outcomes in CRC [29]. Currently, the gold standard for CRC staging relies on the tumor–node–metastasis (TNM) classification [30, 31]. This assessment typically requires invasive diagnostic procedures, including colonoscopy and tissue biopsy, followed by detailed pathological evaluation [32, 33]. In light of these limitations, serum metabolic profiling could serve as a time‐efficient and minimally invasive tool with potential advantages over conventional CRC screening methods [34]. Importantly, the high–dimensional nature of metabolomic data makes it particularly well‐suited for machine learning–based analytical frameworks. However, clinical prognostic assessment has traditionally relied on individual circulating biomarkers, such as C‐reactive protein–to–albumin ratio, rather than integrative metabolite‐based models [35]. Consequently, machine learning approaches that integrate circulating metabolite information for disease stratification and prognosis remain relatively underutilized.

To address these challenges, we applied a ^1^ ^9^F NMR–based amine profiling strategy for quantitative analysis of serum amino acids. This approach uses fluorinated probes that selectively bind to primary amines, generating distinct and quantifiable ^1^ ^9^F NMR signals for amino acids with minimal interference from unrelated serum components [36, 37, 38]. The method enables accurate and reproducible metabolite quantification in liquid biospecimens such as serum. To improve analytical robustness, we implemented a ratio–based normalization strategy designed to mitigate inter–cohort variability and capture coordinated metabolic shifts across the amino acid network [39, 40, 41]. We further integrated network–level metabolite features with machine learning to evaluate their ability to classify disease stage and predict clinically relevant outcomes, such as recurrence or metastasis. Recognizing the clinical significance of systemic profiling, we also developed a machine learning–based model to predict recurrence or metastasis, critical for post–treatment surveillance that is currently underexplored. Here, we present a ^1^ ^9^F NMR–based amino acid profiling strategy combined with correlation‐based network analysis, suggesting that circulating amino acid networks capture systemic metabolic adaptations during CRC progression and enable improved prediction of disease recurrence and metastasis.

## 2 Results

### 2.1 Workflow of ^1^ ^9^F NMR–Based Serum Profiling and Data Analysis

**FIGURE 1:**
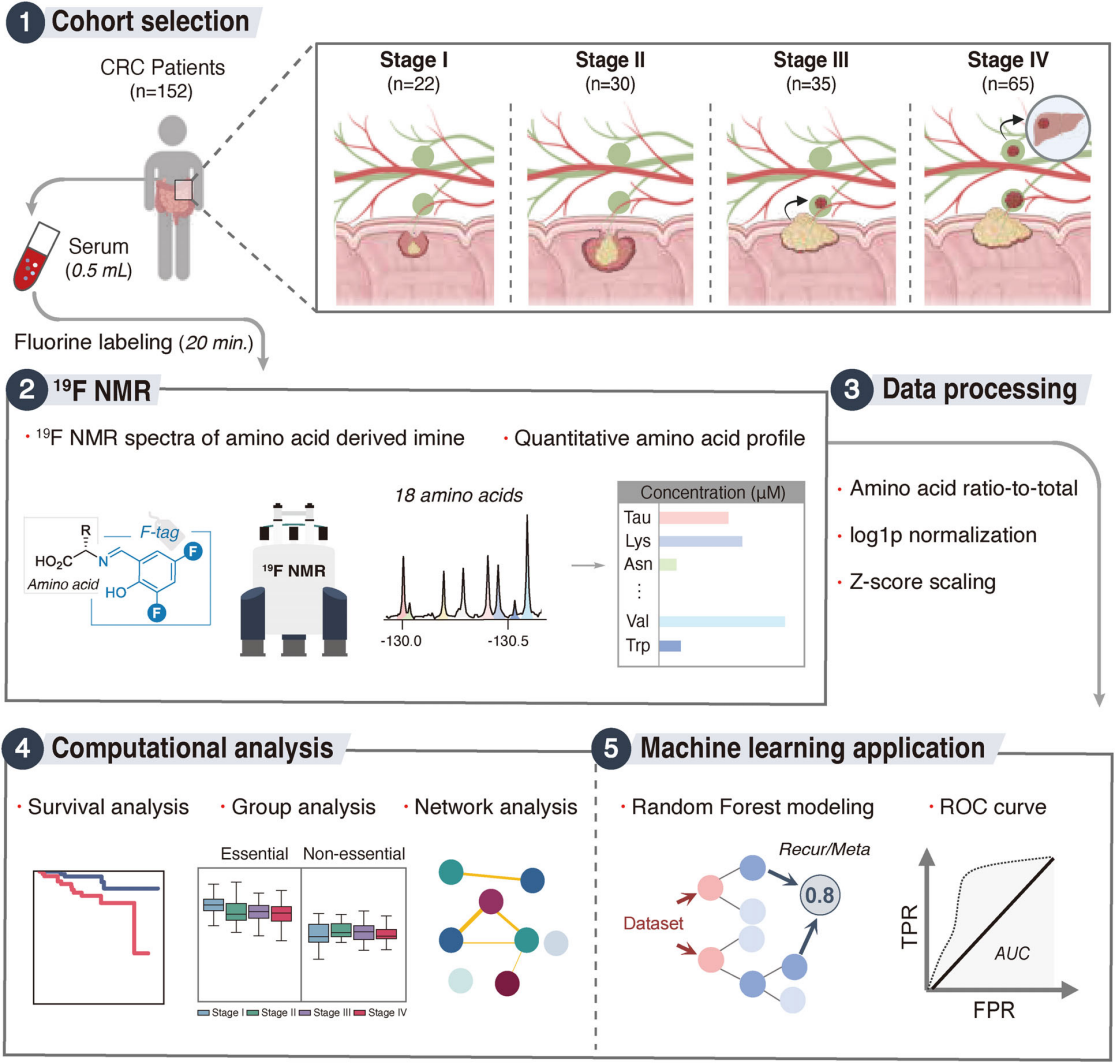
Workflow of ^1^ ^9^F NMR–based serum amino acid profiling and data analysis. Serum samples collected from CRC patients were subjected to fluorine‐labeling–based amino acid detection followed by ^1^ ^9^F NMR spectroscopy. Quantified amino acid data were processed using ratio‐based normalization and multivariate analyses to characterize stage‐specific metabolic patterns and construct machine learning models for prognostic risk prediction.

To investigate stage‐specific alterations in circulating amino acid metabolism during CRC progression, we established a ^1^ ^9^F NMR–based serum amino acid profiling workflow and analyzed preoperative serum samples from 152 CRC patients (Figure 1). Serum samples were collected immediately prior to surgery, following standardized fasting and bowel preparation conditions. Clinical variables, including sex, age, body mass index (BMI), and total amino acid concentration, did not differ significantly across tumor stages (Figure S1A), indicating that the observed changes in amino acid profiles represent stage‐specific metabolic signatures rather than confounding effects. Baseline demographic and clinical characteristics of the cohort are summarized in Table S1.

Using fluorine‐based amine labeling, ^1^ ^9^F NMR spectroscopy enabled simultaneous detection and quantification of 18 amino acids within a single spectrum (Figure S1B). This analytical platform enabled robust serum amino acid quantification and supported both metabolite‐level profiling and interaction‐based analysis of amino acid relationships across CRC stages. This approach facilitated a ratio‐based normalization strategy, which minimized potential artifacts from experimental variation. Low‐abundance amino acids, including values at or below the detection limit, were handled using log1p transformation to accommodate zero values while preserving relative relationships among amino acids (Figure S1C). To analyze stage‐specific differences in amino acid profiles, Z‐score scaling was optionally applied depending on the analytical objective.

Finally, we performed machine learning‐based analyses using serum amino acid profiles to predict cancer recurrence or metastasis and evaluated their prognostic performance in comparison with the conventional biomarker carcinoembryonic antigen (CEA). While conventional metabolomic analyses rely on absolute metabolite concentrations that may limit cross‐cohort applicability, our framework integrates normalization and interaction‐based features to capture relative metabolic shifts. The integrated model combining individual amino acid levels and correlation‐based features demonstrated improved predictive performance compared with models based on each feature type alone, highlighting the importance of amino acid interaction patterns associated with recurrence and metastasis.

### 2.2 Stage‐Specific Changes in Serum Amino Acid Profiles

**FIGURE 2:**
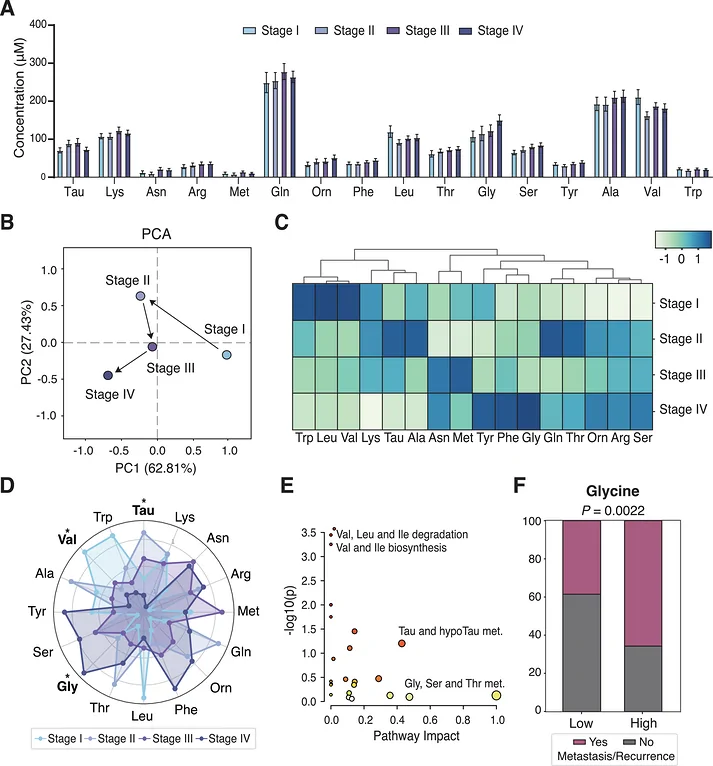
Stage‐specific remodeling of circulating amino acids during CRC progression. (A) Quantification of amino acid concentration detected in serum of CRC patients using ^1^ ^9^F NMR spectroscopy. Data are means ± SEMs from 152 patients (I: *n*=22, II: *n*=30, III: *n*=35, IV: *n*=65). (B) PCA illustrating stage‐related shifts in amino acid profiles. Each dot represents the mean amino acid profile of patients within the corresponding TNM stage. (C) Heatmap visualization of ratio‐normalized amino acid levels across tumor stages, highlighting stage‐dependent metabolic patterns. (D) Radar chart of amino acid profiles by TNM stage. Statistical significance across TNM stages was assessed using the Kruskal–Wallis test. *p < 0.05. (E) Pathway enrichment analysis using MetaboAnalyst to compare stage I and IV. (F) Bar plot of recurrence or metastasis incidence based on serum glycine proportion. Patients were divided into high and low groups based on the median glycine proportion. Statistical significance was assessed using a two‐sided Fisher's exact test.

We first quantified the absolute concentrations of 18 amino acids in CRC patient serum using ^1^ ^9^F NMR spectroscopy (Figure 2A). While absolute concentrations provided a foundation for quantification, they did not reveal statistically significant differences across stages for all individual amino acids. To address this limitation, we applied a ratio‐based normalization strategy followed by log1p and Z‐score transformation, enabling the detection of coordinated metabolic changes across tumor stages. With normalized amino acid profiles, principal component analysis (PCA) revealed a gradual stage‐dependent shift across the CRC cohort (Figure 2B). Heatmap visualization further highlighted distinct patterns of metabolite abundance across TNM stages (Figure 2C). Based on ratio‐normalized levels, amino acids formed three distinct clusters with similar stage‐dependent trajectories (Figure S2A and Table S2). Notably, amino acids with shared biochemical functions tended to cluster together. Cluster 1 comprised branched‐chain amino acids (BCAAs), while Cluster 2 included glycine, serine, and threonine, which are key metabolites involved in the one‐carbon metabolism (Figure S2A). The clustering patterns suggest that ^1^ ^9^F NMR–based serum amino acid profiling effectively preserves biologically meaningful metabolic relationships. Among the amino acids, the ratio‐normalized levels of valine, glycine, and taurine varied significantly across TNM stages (Figure 2D). Valine and glycine exhibited opposite trends during cancer progression: valine levels gradually decreased during disease progression, whereas glycine levels steadily increased. Taurine demonstrated a distinct oscillatory pattern, peaking at stage II and gradually declining in later stages (Figure S2B). Through metabolite set enrichment analysis (MSEA), we confirmed that the metabolic pathways related to those amino acids were substantially altered between stage I and stage IV (Figure 2E). Importantly, ratio‐normalized analysis identified glycine as the amino acid most strongly associated with recurrence and metastasis (Figure 2F and Figure S2C), suggesting that glycine‐associated metabolic remodeling may represent a clinically relevant feature of systemic metabolic adaptation during CRC progression.

### 2.3 Progressive Decline of Essential Amino Acids in Serum During CRC Progression

**FIGURE 3:**
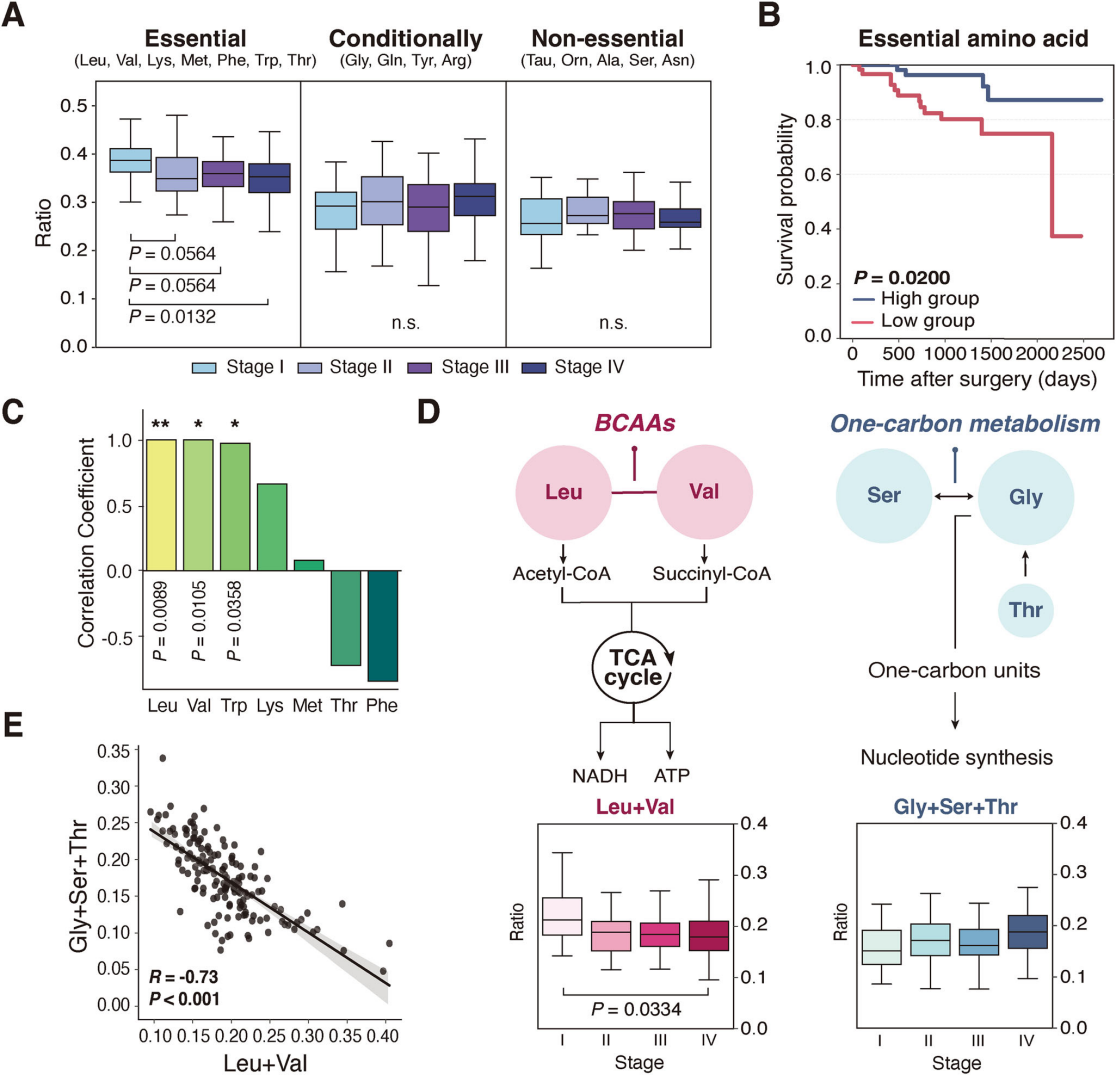
Stage‐dependent redistribution of circulating amino acid classes during CRC progression. (A) Box plots depicting the proportion of essential (EAAs), non‐essential (NEAAs), and conditionally essential amino acids relative to the total across stages. Features significant by the Kruskal–Wallis test (*p* < 0.05) were further analyzed using Dunn's post hoc test with Benjamini–Hochberg FDR correction. (B) Kaplan–Meier curve showing survival probability based on EAAs proportion. Patients were stratified into high‐ and low‐EAA groups based on the median EAA proportion. Statistical significance was assessed using the log‐rank test. (C) Correlation coefficient plot analyzing amino acids correlated with the EAAs ratio. Pearson correlation coefficients were calculated, and statistical significance was assessed using a two‐sided Student's t‐test. (D) Schematic illustrating the stage‐associated metabolic shift between BCAA‐related metabolism and serine–glycine–threonine–linked one‐carbon metabolism. Box plots show stage‐dependent changes in each metabolic axis. Features significant by the Kruskal–Wallis test (*p* < 0.05) were further analyzed using Dunn's post hoc test with Benjamini–Hochberg FDR correction. (E) Scatter plot showing the negative correlation between BCAA levels (leucine + valine) and the combined (glycine + serine + threonine) ratio. Pearson correlation coefficients (*R*) were calculated, with significance assessed using a two‐sided Student's t‐test. Shaded areas indicate 95% confidence intervals.

To further investigate systemic metabolic changes associated with CRC progression, we examined stage‐dependent alterations in the relative composition of circulating amino acids. Amino acids can be further classified by nutritional necessity into essential amino acids (EAAs), non‐essential amino acids (NEAAs), and conditionally essential amino acids [42], as illustrated in Figure S3A. Given that cancer cells selectively consume or secrete specific amino acids, we hypothesized that the systemic balance among different amino acid groups might shift during cancer progression. We therefore examined stage‐specific changes in the relative proportions of circulating amino acids categorized as EAAs, NEAAs, and conditionally essential amino acids. Remarkably, only EAAs exhibited a significant alteration in their relative proportions, progressively declining with CRC progression (Figure 3A). This progressive depletion of EAAs may reflect increased metabolic demands during tumor development. We further found that patients with a lower proportion of EAAs in preoperative serum, stratified by the cohort median, exhibited significantly poorer postoperative survival in our CRC cohort (Figure 3B), suggesting the prognostic relevance of serum EAA depletion in CRC.

Pearson correlation analysis revealed that this gradually decreasing trend in EAAs proportion by stage progression was primarily driven by leucine, valine, and tryptophan (Figure 3C). Valine and leucine were the most and third‐most abundant EAAs by proportion in serum, respectively (Figure S3B), and both are BCAAs, which regulate many key signaling pathways [43]. BCAAs were catabolized through branched‐chain amino acid aminotransferase (BCAT) and branched‐chain α‐keto acid dehydrogenase complex (BCKDH), yielding acetyl‐coenzyme A (acetyl‐CoA) from leucine and succinyl‐coenzyme A (succinyl‐CoA) from valine via propionyl‐coenzyme A (propionyl‐CoA), which entered the tricarboxylic acid (TCA) cycle [44, 45]. This process promoted adenosine triphosphate (ATP) synthesis through nicotinamide adenine dinucleotide (NADH) and flavin adenine dinucleotide (FADH_2_) production [46]. Given that glycine showed stage‐dependent elevation and prognostic relevance in Figure 2, we next focused on the glycine‐centered one‐carbon metabolic axis. Within this axis, serine can be converted into glycine and one‐carbon units that contribute to nucleotide biosynthesis [47, 48], and threonine can also be metabolized into glycine [49, 50].

Consistent with this analysis, circulating leucine and valine—BCAAs that contribute to TCA cycle metabolism—decreased in a stage‐dependent manner, whereas serine, glycine, and threonine—amino acids linked to one‐carbon metabolic pathways—increased during CRC progression (Figure 3D). Consistent with these opposing stage‐dependent trends, correlation analysis showed that circulating BCAA levels were significantly and negatively correlated with serine, glycine, and threonine across CRC progression (Figure 3E).

To determine whether these alterations in circulating amino acid profiles were evident in early‐stage CRC in an independent cohort, we analyzed an external plasma cohort from Asan Medical Center (AMC), including healthy controls, stage I patients, and stage I–IV patients (Figure S4). No significant differences were observed among the groups in terms of sex distribution, age, BMI, or total amino acid levels (Figure S4A). The data were processed using the same preprocessing and analytical pipeline as applied to the serum‐based dataset. Consistent with the primary analysis, the relative proportion of EAAs progressively decreased in stage I and stage I–IV patients compared with healthy controls. In contrast, NEAAs showed an increasing trend in the same comparison, a pattern newly observed in this external cohort (Figure S4B). Among EAAs, valine showed the strongest association, whereas taurine was identified as a major contributor to the NEAA increase (Figure S4C). The relative proportions of the measured BCAA components, leucine and valine, also progressively decreased from healthy controls to Stage I and Stage I–IV patients. In contrast, the ratio of one‐carbon metabolism–related amino acids, including glycine, serine, and threonine, remained relatively stable across these groups (Figure S4D).

Furthermore, stage‐stratified correlation analysis in the external plasma cohort recapitulated the interaction patterns of key amino acids identified in the serum dataset, including valine and leucine. In particular, BCAA‐centered correlation changes and glycine‐related interaction patterns were preserved across TNM stages (Figure S4E). These findings suggest that the observed metabolic network remodeling is not restricted to serum samples but reflects a shared alteration in circulating amino acid profiles associated with CRC progression.

### 2.4 Remodeling of Amino Acid Interaction Networks and Prediction of Recurrence or Metastasis

**FIGURE 4:**
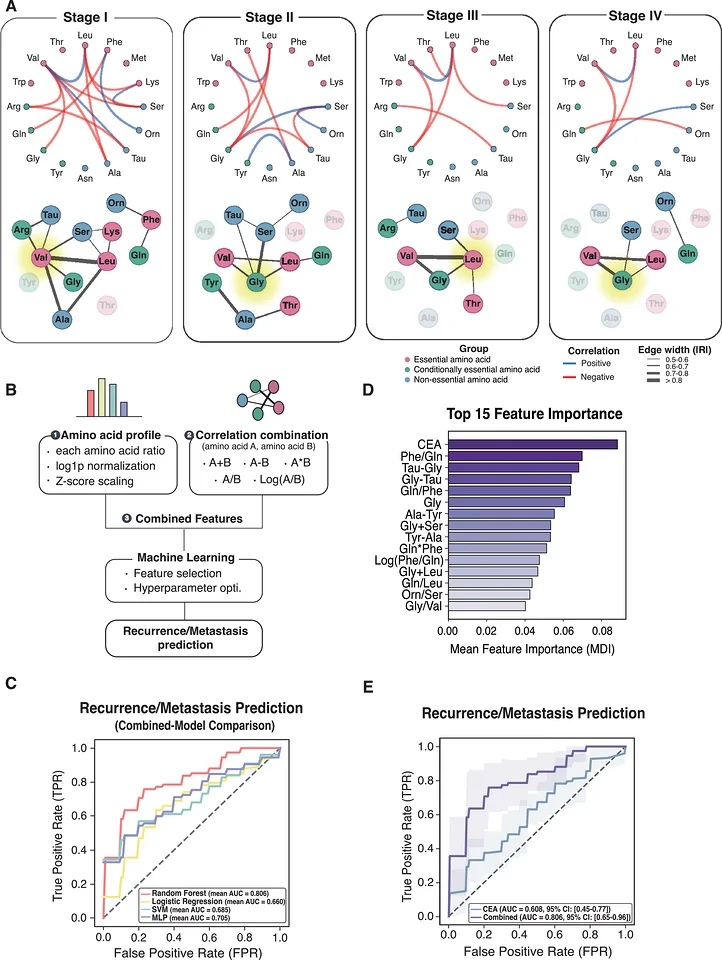
Dynamics in amino acid interactions by TNM stages. (A) Network visualization of amino acid correlations across TNM stages. Essential (pink), conditionally essential (green), and non‐essential amino acids (blue) with statistically significant interactions are demonstrated. Blue edges indicate positive correlations, whereas red edges indicate negative correlations. Edge width represents the strength of the correlation. (Pearson correlation |*R*| > 0.50 and a two‐sided Student's t‐test *p* < 0.05) (B) Workflow for recurrence or metastasis prediction using serum amino acid profiles. After transformation and normalization, three models were developed: amino acid–based, correlation‐based (pairwise features), and a combined model with CEA. A machine learning model with nested cross‐validation was used for feature selection and prediction. (C) Receiver operating characteristic (ROC) curves comparing the performance of different machine learning models for recurrence or metastasis prediction using the combined feature set. Models include Random Forest, Logistic Regression, Support Vector Machine (SVM), and Multi‐layer Perceptron (MLP). Each curve represents the mean ROC across outer folds in nested cross‐validation, with shaded regions indicating variability across folds. The Random Forest model achieved the highest overall performance among the evaluated models. (D) Top 15 ranked by importance (mean decrease in impurity, MDI) in the combined Random Forest model predicting recurrence or metastasis. The *x*‐axis shows importance values, reflecting each feature's contribution to classification. (E) ROC curve showing recurrence or metastasis prediction performance using the amino acid profile dataset. Mean AUROC and standard deviation were obtained from nested cross‐validation (outer 7 folds), and the shaded area indicates the 95% CI across folds.

While analyses of individual metabolite levels revealed stage‐dependent changes in circulating amino acids, metabolic processes operate through coordinated interactions among metabolites. To investigate stage‐specific metabolic dynamics in CRC [51, 52], we profiled pairwise relationships among amino acids rather than focusing on individual metabolites. This panel‐based approach revealed interaction‐level alterations that were not apparent from individual concentrations alone. Early‐stage tumors exhibited relatively dense interaction patterns among amino acids, whereas advanced stages showed reduced overall connectivity (Figure S5A). This pattern suggests that the initially dense amino acid networks are disrupted during cancer progression, leaving fewer than half of the pairs significantly correlated. Among the amino acids analyzed, valine displayed the highest degree of connectivity in early‐stage networks, interacting with multiple metabolites, including alanine, taurine, and serine (Figure 4A). However, these interactions progressively weakened as CRC advanced. In contrast, correlations involving glycine became increasingly prominent, suggesting that glycine‐associated metabolic relationships are selectively maintained or strengthened during disease progression. The decrease in the number of significant valine‐associated correlation pairs aligned with the stage‐dependent decline in circulating valine levels (Figure S2B), suggesting that reductions in valine contributed to the loss of several metabolic interactions. In contrast, correlations between valine and glycine, as well as valine and leucine, were strengthened. Similarly, a reduction in circulating taurine levels from stage II to stage III coincided with a decrease in taurine‐associated correlation pairs (Figure 4A). In stage III, the negative correlation between valine and glycine became markedly stronger and persisted into stage IV. In contrast, the positive correlation between valine and leucine weakened in stage IV, whereas a positive relationship between serine and glycine re‐emerged. Together, these dynamic changes illustrate progressive restructuring of circulating amino acid interaction networks during CRC progression.

In the previous section, we observed that the relative proportion of BCAAs (i.e., leucine and valine) decreased with advancing tumor stage, whereas the proportion of one‐carbon metabolism‐related amino acids such as glycine, serine, and threonine increased during CRC progression [53]. Consistent with these compositional changes, glycine and BCAAs maintained strong negative correlations across stages. In contrast, serine and glycine, amino acids associated with one‐carbon metabolism critical for cancer cell survival [54, 55], re‐established a positive correlation at stage IV.

We next developed a machine learning framework to predict clinically relevant disease progression using serum amino acid profiles. To capture broader prognostic risk, the primary prediction target was defined as a composite endpoint of recurrence or metastasis. In this endpoint definition, stage IV patients were classified as metastatic at baseline, whereas stage I–III patients were classified according to follow‐up‐derived recurrence status. Patients in stages I–III without available recurrence information were excluded from the analysis.

We constructed three types of models (Figure 4B): (1) an amino acid–based model, which used individual amino acid concentrations as input features; (2) a correlation‐based model, which incorporated pairwise interaction features derived from statistically significant amino acid correlations (sum, difference, product, ratio, and log‐ratio); and (3) a combined model, which integrated both feature types along with CEA. All models were trained using a nested cross‐validation framework with 7 outer folds and 4 inner folds, with feature selection and hyperparameter optimization performed within each inner fold.

To further evaluate the robustness of our modeling framework, we compared multiple machine learning algorithms, including logistic regression, support vector machines (SVM), and multilayer perceptron (MLP), under identical nested cross‐validation settings (Figure 4C). Across all models, Random Forest consistently demonstrated the best predictive performance (Table S3). In the combined feature setting, it achieved a mean AUROC of 0.806 ± 0.081, outperforming logistic regression (0.660 ± 0.050), SVM (0.685 ± 0.191), and MLP (0.705 ± 0.116). Notably, Random Forest also exhibited a well‐balanced classification profile, with a sensitivity of 0.727 and a specificity of 0.697. Furthermore, feature importance analysis revealed distinct differences in feature utilization across models. Random Forest incorporated a broad range of amino acid interaction features, effectively capturing correlation‐based features in a distributed and global manner. In contrast, logistic regression, SVM, and MLP tended to rely on a more limited subset of features (Figure S5B). Based on these results, the Random Forest model was selected as the primary predictive model for recurrence or metastasis.

In the amino acid–based model, glycine emerged as the most predictive feature (Figure S5C), consistent with our earlier findings that recurrence or metastasis status was significantly associated with glycine abundance (Figure 2F and Figure S2C). In the correlation‐based model (Figure S5D), several glycine‐involving amino acid pairs—such as glycine–taurine, glycine–serine, and glycine–leucine—ranked among the top predictors, with glycine–taurine placing first in overall feature importance. Additionally, pairs involving leucine (a BCAA), such as glutamine–leucine and glycine–leucine, were also among the top‐ranked features. Although valine–glycine ranked lower than the top glycine‐involving pairs, it was still retained among the selected correlation‐derived features.

Subsequently, we constructed a combined model by integrating individual amino acid features, correlation‐derived features, and the CEA marker. In this model, CEA ranked as the most important feature (Figure 4D), reconfirming its conventional role as a tumor‐associated antigen biomarker. Notably, among the top 15 features, excluding CEA, the majority were associated with BCAAs such as valine and leucine, as well as serine and glycine, highlighting the pivotal role of amino acid–based features in predicting recurrence or metastasis.

Performance comparison revealed a stepwise improvement across models: CEA model yielded an AUROC of 0.608 ± 0.082 (95% CI: 0.447–0.770), amino acid–based model at 0.694 ± 0.106 (95% CI: 0.485–0.902), the correlation‐based model at 0.759 ± 0.081 (95% CI: 0.599–0.918), and combined model at 0.806 ± 0.081 (95% CI: 0.648–0.964). In addition to AUROC, we systematically evaluated classification performance using accuracy, sensitivity, specificity, precision, and confusion matrices to provide a more comprehensive assessment of model performance. These additional metrics consistently supported the superiority of the combined model, indicating improved identification of high‐risk patients while reducing unnecessary misclassification of low‐risk cases (Table S4). In terms of classification performance at the optimal threshold, the combined model achieved the highest sensitivity and specificity (0.727 and 0.697, respectively), outperforming CEA (0.565 / 0.614), amino acid‐based (0.712 / 0.521), and correlation‐based (0.701 / 0.700) models (Figure S4E). Consistent with these findings, confusion matrix analysis further demonstrated improved classification performance of the combined model, with reduced false negatives and false positives compared to the CEA‐only model (Figure S5F). This indicates a more clinically balanced prediction performance, improving both sensitivity and specificity without sacrificing either metric. This progression highlights the predictive value of correlation‐derived features and the synergistic improvement achieved when integrated with the conventional CEA marker.

To further determine whether the composite endpoint reflected shared or endpoint‐specific metabolic signals, we performed additional analyses by separating the prediction tasks into recurrence‐only and metastasis‐only settings. Stage IV patients with recurrence were excluded to ensure a strict definition of metastasis‐only cases (Figure S6A,B). Despite the reduced sample size, correlation‐based features showed the highest predictive performance in both endpoint‐specific tasks, with mean AUROCs of 0.824 for recurrence‐only prediction and 0.796 for metastasis‐only prediction (Figure S6C,D and Table S5). In recurrence‐only prediction, the correlation‐based model outperformed the CEA‐only model (AUROC = 0.476) and the amino acid–based model (AUROC = 0.647). Similarly, in metastasis‐only prediction, the correlation‐based model showed higher performance than the CEA‐only model (AUROC = 0.569) and the amino acid–based model (AUROC = 0.560). Feature importance analysis identified distinct endpoint‐specific patterns. The recurrence‐only model included interactions involving leucine, glutamine, and phenylalanine, whereas the metastasis‐only model included features associated with glycine, taurine, and alanine (Figure S6E,F). Notably, these endpoint‐specific metabolites were also represented among the important features in the combined model (Figure 4D). These findings suggest that the composite endpoint model captures clinically relevant progression signals while preserving partially distinct metabolic patterns associated with recurrence and metastasis.

## 3 Discussion

This study demonstrates that circulating amino acid networks undergo progressive remodeling during CRC progression. Using a ^1^ ^9^F NMR–based serum profiling platform, we quantified 18 amino acids in patient‐derived serum. We then used a ratio‐based normalization strategy to capture both individual metabolite dynamics and interaction‐level metabolic relationships across tumor stages. Stage‐dependent metabolic alterations were characterized by depletion of BCAAs, including valine and leucine, together with increased abundance of metabolites such as glycine. These opposing trends suggest that tumor progression is associated with shifts in amino acid utilization and systemic metabolic redistribution. Notably, glycine consistently emerged as a central metabolite linking metabolic alterations with clinical outcomes. This suggests that glycine‐associated metabolic rewiring may represent a key feature of systemic metabolic adaptation during CRC progression.

Because BCAAs are essential amino acids that cannot be synthesized *de novo*, the stage‐dependent decline in valine and leucine may reflect increased metabolic demand during tumor progression [56, 57]. Moreover, depletion of circulating BCAAs may contribute to systemic metabolic imbalance associated with cancer cachexia, a condition frequently observed in advanced‐stage patients [58, 59].

In contrast to BCAA depletion, circulating glycine increased in patients with advanced CRC despite its involvement in one‐carbon metabolism. This apparent paradox may reflect compensatory host responses rather than reduced tumor utilization. Unlike BCAAs, glycine and serine are non‐essential amino acids that can be synthesized *de novo* in host organs, including the liver, kidney, and skeletal muscle [47, 60]. Sustained tumor‐associated demand for one‐carbon metabolism may create an imbalance between tumor and host metabolism, promoting compensatory synthesis and release of serine and glycine by host tissues to maintain systemic homeostasis [61, 62]. In this context, elevated circulating glycine may represent a systemic marker of tumor–host metabolic crosstalk. Similar observations have also been reported in other cancers [63, 64, 65], suggesting that this phenomenon may represent a broader feature of tumor–host metabolic interactions. Together, these findings suggest that BCAA depletion and increased levels of one‐carbon metabolism–related amino acids represent distinct but coordinated components of systemic amino acid network remodeling during CRC progression.

These serum‐based findings were further supported by the external AMC plasma cohort, which included healthy controls, Stage I patients, and Stage I–IV patients. In this independent plasma cohort, the relative proportions of EAAs and BCAA components progressively decreased from healthy controls to Stage I and Stage I–IV patients, suggesting that circulating amino acid remodeling may already be detectable at early stages of CRC. In contrast, the relative abundance of glycine, serine, and threonine showed relatively limited changes across these groups, suggesting that one‐carbon metabolism–related amino acid alterations may become more prominent during disease progression rather than during initial tumor development. These findings further support that circulating amino acid remodeling is not restricted to the primary serum cohort and can also be captured across different blood‐derived sample types.

To further examine whether CRC cells can directly reshape extracellular amino acid composition, we performed ^1^ ^9^F NMR–based amino acid profiling of conditioned media from CRC cell lines and a normal colon epithelial cell line (Figure S7A–E). Ratio‐based analysis of time‐dependent changes in extracellular amino acid composition revealed that CRC cell lines exhibited more dynamic remodeling of the extracellular amino acid environment than the HCEC normal colon epithelial cell line. These findings suggest that CRC cells can actively alter their surrounding amino acid environment, providing supportive experimental evidence that tumor cell–intrinsic metabolism may contribute, at least in part, to the circulating amino acid alterations observed in patient serum.

Beyond changes in individual metabolite abundance, correlation analysis revealed substantial reorganization of circulating amino acid interaction networks during CRC progression. In particular, BCAA‐related networks centered on valine and leucine were progressively weakened during cancer progression, whereas glycine‐centered interaction networks became relatively more prominent. These interaction‐level alterations were not apparent from absolute metabolite concentrations alone, highlighting the importance of network‐based analyses for capturing systemic metabolic states during cancer progression.

We further evaluated the clinical relevance of these network‐level metabolic features using machine learning models derived from serum amino acid profiles. Glycine emerged as the most predictive feature in the amino acid–based model, and glycine‐centered interaction pairs ranked among the most important predictors in the correlation‐based model. These findings suggest that glycine‐centered metabolic interactions not only reflect network remodeling associated with CRC progression but may also contribute to predicting recurrence or metastasis risk. When recurrence and metastasis were modeled as separate endpoints, feature importance analysis revealed distinct amino acid interaction patterns between the two outcomes. While recurrence‐associated features were enriched for leucine‐, glutamine‐, and phenylalanine‐related interactions, metastasis‐associated features were characterized by glycine‐, taurine‐, and alanine‐centered interactions. These differences suggest that recurrence and metastasis may be driven by partially distinct metabolic programs, despite both representing disease progression. Importantly, the model predicting recurrence or metastasis as a composite endpoint incorporated representative features from each endpoint‐specific model, indicating that it captured mixed metabolic patterns from both clinically distinct outcomes. Thus, the combined model can be interpreted as a unified predictive framework that integrates multiple endpoint‐specific metabolic patterns rather than obscuring biological heterogeneity.

Consistent with the predictive value of network‐level features, integration of correlation‐derived interaction features substantially improved predictive performance in Random Forest models, achieving the highest performance when combined with the conventional biomarker CEA (AUROC = 0.806 ± 0.081). Clinically, CEA showed relatively high specificity but limited sensitivity for identifying patients at high risk of recurrence or metastasis, whereas amino acid–derived metabolic features improved sensitivity for detecting metabolically active disease states. Together, these findings suggest that conventional CEA and amino acid–derived metabolic features provide complementary information for identifying patients at risk of recurrence or metastasis.

Despite these strengths, several limitations should be acknowledged. First, the current ^1^ ^9^F NMR protocol did not capture the full set of 20 amino acids; cysteine, aspartic acid, histidine, and proline were not quantified. Second, spectral overlap between isoleucine and glutamate prevented reliable quantification of their individual concentrations. Third, our analysis focused primarily on amino acids related to representative metabolic pathways implicated in cancer progression, including the TCA cycle and one‐carbon metabolism. Future studies incorporating amino acids and related metabolites from broader metabolic pathways may provide a more comprehensive understanding of metabolic remodeling during CRC progression. Fourth, this study is retrospective and based on a single‐center cohort, and the patient distribution is relatively enriched for stage IV disease, which may limit the generalizability of our findings. Finally, patients were classified into broad stages (I–IV) rather than detailed TNM subcategories. Future studies based on larger, prospective, multi‐center cohorts with more balanced stage distributions and optimized analytical protocols are warranted to validate and extend our findings.

In summary, circulating amino acid networks undergo progressive remodeling during CRC progression. By combining ^1^ ^9^F NMR–based serum amino acid quantification with correlation‐based network analysis and machine learning modeling, this study provides a minimally invasive strategy for capturing systemic metabolic states associated with tumor progression. Serum amino acid network profiling may therefore serve as a complementary approach for prognostic stratification, postoperative surveillance, and long‐term monitoring in patients with CRC.

## 4 Experimental Section/Methods

### 4.1 Experimental Design

The objective of this study was to evaluate whether serum amino acid profiles reflect stage‐specific systemic metabolic remodeling in colorectal cancer and whether these metabolic features are associated with clinical outcomes. We conducted a retrospective observational study using preoperative serum samples and corresponding clinical records from patients with pathologically confirmed colorectal cancer.

Tumor stage was defined based on pathological assessments recorded in the hospital medical records, and patients were stratified accordingly. Information on recurrence and metastasis was available from clinical follow‐up data at the time of analysis; in this study, these outcomes were specified a priori as primary clinical endpoints.

The overall analytical framework, including comparisons of amino acid concentrations, analyses of pairwise correlation patterns, and construction of machine learning–based predictive models, was defined prior to outcome assessment. During model development, outcome labels from validation and test sets were not used in the training process to minimize information leakage and ensure unbiased performance evaluation.

### 4.2 Clinical Cohort

The study protocol was approved by the Institutional Review Board of Gangnam Severance Hospital (IRB No. 3‐2023‐0429) and KAIST (IRB No. 2024‐06), and all procedures were conducted in accordance with the ethical standards of the Declaration of Helsinki. This retrospective study utilized serum samples collected from 181 CRC patients at Gangnam Severance Hospital between 2015 and 2022. To ensure clinical consistency, a total of 152 patients aged 45–80 years diagnosed with CRC were enrolled, excluding those with cecal cancer. Only cases involving the ascending colon, transverse colon, descending colon, sigmoid colon, and rectum were included. To ensure consistency across serum samples, blood was collected immediately prior to surgery following standardized fasting and bowel preparation protocols. Sample acquisition involved only routine preoperative blood collection, and no procedure‐related issues were reported.

All serum samples were obtained with written informed consent from all participants, and all personal identifiers were removed prior to analysis. Subjects were allocated to groups according to pathological tumor stage (pTNM classification); randomization was not applied. Blinding of investigators or analysts was not applicable, as this study involved retrospective analysis of anonymized clinical data. No formal power calculation was performed; the sample size was determined based on data availability and inclusion criteria. Recurrence or metastasis status was determined independently based on clinical follow‐up records, without reference to serum amino acid profiles or model‐derived scores.

### 4.3 External Validation Cohort (AMC Cohort)

To validate the reproducibility of our findings, we analyzed an independent external cohort using plasma samples collected at Asan Medical Center (AMC). The study protocol was approved by the Institutional Review Board (IRB) of Asan Medical Center (No. 2025‐0948) in accordance with Good Clinical Practice guidelines and the principles of the Declaration of Helsinki. All patient data were anonymized prior to analysis, and the requirement for written informed consent was waived by the IRB. The biospecimens and data used in this study were provided by the Asan Bio‐Resource Center, Korea Biobank Network (2025‐16(317)).

The AMC cohort consisted of 50 healthy controls and 120 colorectal cancer patients, including 30 patients each from Stage I, II, III, and IV. Blood samples from healthy controls were obtained from individuals undergoing routine health check‐ups at the Health Promotion Center of Asan Medical Center, while plasma samples from colorectal cancer patients were obtained from the Asan Bio‐Resource Center.

To ensure comparability between healthy controls and patients, the cohort was constructed with age filtering (45–75 years) to minimize differences in age distribution. As a result, no significant differences were observed among groups in terms of sex, age, body mass index (BMI), or total amino acid levels.

All plasma samples were processed using the same preprocessing and analytical pipeline as applied to the primary serum cohort.

This external cohort was used to validate the robustness and generalizability of amino acid composition, correlation patterns, and metabolic features identified in the primary analysis and was not used for model training.

### 4.4 Amino Acid Profiling Through ^19^F NMR Spectroscopy

For CRC patient serum analysis, sample preparation involved protein precipitation and fluorine‐labeling steps. After thawing 0.5 mL of serum at room temperature, 1 mL of methanol was added to precipitate proteins. After incubating the mixture at −20°C for 20 min, the precipitates were filtered out through a syringe filter (0.2 µm, 25 mm). Any remaining volatiles were completely evaporated using a rotary evaporator. Next, 3,5‐difluoro‐salicylaldehyde (1 mg, 6 µmol) as the fluorine‐tagging reagent was added in 2 mL of methanol, followed by sonication for 20 min to enable fluorine‐tagging. After the reaction, methanol was evaporated, and 0.75 mL of CD_3_OD containing an internal standard (20 µL of 9 mM CD_3_OD stock solution) was added. Samples were then transferred to thin‐wall NMR tubes (Deutero, No. D400‐5‐8).

All ^19^F{^1^H} NMR experiments were performed at 298 K on a Bruker AVANCE III HD (9.4 T) 400 MHz spectrometer equipped with a 60‐slot automatic sample handler and a BBFO 400 MHz S1 5 mm probe with z‐gradient or Bruker AVANCE NEO Nanobay (9.4 T) 400 MHz with iProbe HR Liquids. ^19^F{^1^H} NMR (Inverse‐gated decoupling) spectra were recorded at 376 MHz using the Bruker ‘zgfhigqn’ pulse sequence with the optimized parameters (pulse angle of 90°, 256 scans, a relaxation delay (D1) of 3 s, and an acquisition time of 1.4 s). The total experiment time was 19 min 12 s. ^19^F{^1^H} NMR spectra were processed and analyzed using MestReNova Software (v14.0.1‐23559). Spectra were phased and corrected using manual correction or automatic adjustment (Whittaker Smoother function) to achieve a flat baseline. The signal‐to‐noise ratio (S/N) was increased by the apodization setting (exponential at 1 Hz). The chemical shifts were reported in parts per million (ppm) and referenced to the fluorine signals of internal standard whose NMR signal was set at ‐138.15 ppm. Automated peak detection and integration were performed using the deconvolution tool in Mnova to achieve the best fit to the original spectrum.

^19^F{^1^H} NMR spectra (376 MHz) of the solution were recorded, and the fluorine signals of the Schiff bases of 18 amino acids were identified based on the previous work (Figure S1B). The absolute concentrations of the 18 detected amino acids were calculated based on the concentration of the internal standard in the ^19^F{^1^H} NMR spectra and corrected using the slope and intercept values from the calibration curve. Detailed experimental and analytical protocols, including sample preparation, NMR acquisition, and data preprocessing steps, are described above to ensure reproducibility.

All chemicals were purchased from TCI Chemicals and Sigma‐Aldrich, and used without further purification or drying. Solvents for sample preparation and analysis, such as methanol (MeOH) and methanol‐d4 (CD_3_OD) were purchased from Samchun Chemicals (Korea) and Deutero.

### 4.5 ^19^F NMR Raw Data Preprocessing

We analyzed the distribution of serum amino acid profiling data and found that the peaks of isoleucine (Ile) and glutamate (Glu) frequently overlapped in the ^1^ ^9^F NMR spectra. As a result, these amino acids were excluded from individual amino acid analyses. However, their quantitative values were retained for calculating the total amino acid concentration, as they still contained relevant information.

In this study, the relative abundance of each amino acid was calculated by normalizing its concentration to the total amino acid concentration per patient. This approach allowed us to better capture the dynamic balance between input and output metabolites while minimizing sample‐to‐sample variation introduced by experimental handling.

Certain amino acids—such as asparagine (Asn), arginine (Arg), methionine (Met), and tryptophan (Trp)—exhibited low detection frequencies or highly skewed distributions in the ^1^ ^9^F NMR spectra (Figure S1C). To address this, we applied a log1p transformation to each amino acid, which preserved the interpretability of zero values while correcting distributional imbalance. In contrast, the remaining amino acids showed relatively uniform distributions and fell within the detectable range of the NMR method.

To enable feature comparability and downstream analysis, Z‐score normalization was subsequently applied to the entire amino acid dataset. This transformation facilitated statistical modeling by mitigating distributional heterogeneity.

### 4.6 Prognostic Model Development for Recurrence or Metastasis

A predictive machine learning model was developed to assess the risk of recurrence or metastasis in CRC patients using serum amino acid profiles. Serum concentrations of 16 amino acids were quantified using nuclear magnetic resonance (NMR) spectroscopy. Ratio‐based normalization was applied to obtain relative amino acid abundances. To reduce data skewness and maintain interpretability of zero values, data were transformed using log1p and subsequently standardized using Z‐score normalization.

The outcome variable, defined as recurrence or metastasis occurrence (Meta_or_Recur), was converted into a binary variable (1 for “Yes” and 0 for “No”). Recurrence or metastasis was determined based on pathological and radiological assessments documented in official clinical records.

For clarity, the composite outcome was defined as follows: patients with stage IV disease were classified as metastatic at baseline, reflecting clinically confirmed distant organ involvement. For patients with stage I–III disease, recurrence status was determined based on longitudinal clinical follow‐up records, and patients with documented recurrence were classified as positive events. Patients with stage I–III disease without available or reliable recurrence information were excluded to ensure accurate outcome labeling.

The criteria for defining recurrence and metastasis were pre‐specified prior to data analysis. Clinically confirmed recurrence or metastasis was selected as the reference standard and used as the prediction target (outcome) for the model. The final analysis cohort for the composite outcome included 140 patients.

In addition to the composite outcome, endpoint‐specific analyses were conducted by constructing separate models for recurrence‐only and metastasis‐only prediction. The recurrence‐only model was restricted to patients with stage I–III disease, with recurrence defined based on longitudinal clinical follow‐up. For the metastasis‐only model, only stage IV patients without recurrence were included as positive cases to ensure clear separation from recurrence events. Stage I–III patients without recurrence were used as negative controls. Stage IV patients who also experienced recurrence were excluded from this analysis.

Four feature sets were constructed for model development:

1. CEA Feature Set: Preoperative carcinoembryonic antigen (CEA) levels.
2. Amino Acid (AA) Feature Set: Z‐score‐normalized serum levels of 16 amino acids.
3. Correlation‐based Feature Set: Correlation features were created using amino acid pairs selected based on Pearson correlation analysis performed within each pTNM stage (stage I‐IV). Only pairs with an absolute correlation coefficient (|*R*|) ≥ 0.50 and two‐sided Student's t test *p* < 0.05 in at least one stage were retained. For these statistically significant amino acid pairs, interaction terms were computed including the arithmetic sum, signed differences (A–B and B–A), ε‐adjusted product ((A+ε)×(B+ε)), raw ratios ((A+ε)/(B+ε) and (B+ε)/(A+ε)), and reciprocal log‐ratios (log((A+ε)/(B+ε)) and log((B+ε)/(A+ε)), ε = 1×10^−^^8^). These interactions were computed only for pairs present within the AA feature set. All interaction features were combined into the correlation‐based interaction matrix (X_corr).
4. Combined Feature Set: Integration of AA, correlation‐based interactions (X_corr), and CEA features into a single combined matrix (X_combined). Patient indices were precisely aligned across feature matrices to ensure data integrity.

Data alignment ensured the inclusion of only samples with complete feature measurements. Infinite values resulting from feature calculations were replaced with NaN and removed from subsequent analyses.

Nested cross‐validation (CV) was employed to develop and validate the model rigorously, using a 7‐fold outer CV for unbiased model evaluation and a 4‐fold inner CV for hyperparameter tuning and feature selection. A fixed random seed (42) was applied throughout to ensure reproducibility. The final decision threshold for classification was derived using Youden's J statistic in an exploratory manner and was not pre‐specified prior to analysis.

For endpoint‐specific analyses (recurrence‐only and metastasis‐only models), the nested CV scheme was adjusted to 5‐fold outer CV and a threefold inner CV due to reduced sample sizes.

All preprocessing steps—including NMR quantification, normalization, and interaction feature construction—were conducted without access to recurrence or metastasis outcomes. Clinical outcome labels were used only during supervised model training, while evaluation was performed on held‐out outer folds to avoid information leakage.

For the CEA model, no feature selection was necessary due to a single‐feature scenario. For all other feature sets, a preliminary Random Forest classifier was trained on the full training set to rank features based on importance (mean decrease in impurity). Candidate features for further analysis were selected based on cumulative importance rankings. Inner CV was then conducted to determine the optimal number (k) of top‐ranked features maximizing the average area under the receiver operating characteristic curve (AUROC).

Hyperparameter tuning was performed via HalvingGridSearchCV with an iterative strategy, using the number of estimators as the adaptive resource. The parameter grid included: max_depth (None, 10, 20, 30), min_samples_split (2, 5, 10), min_samples_leaf (1, 2, 4), max_samples (None, 0.8, 0.9), max_features (“sqrt”, “log2”, 0.4, 0.6, 0.8), bootstrap (True), and class_weight (“balanced”).

Model performance was assessed on outer CV folds. ROC curves were plotted, and the optimal classification threshold was derived using Youden's J statistic with sensitivity‐weighted optimization. Performance metrics, including AUROC, sensitivity, specificity, and precision‐recall area under the curve (PR AUC), as well as accuracy, precision, and F1 score, were calculated. Confusion matrices were generated by aggregating true negatives, false positives, false negatives, and true positives across outer CV folds for each feature set. Mean true positive rate (TPR) curves were interpolated on a fixed false positive rate (FPR) grid (0 to 1, 101 points), and average ROC curves were computed along with ±1 standard deviation across CV folds. All computational procedures were implemented using Python (v3.11.11; RRID:SCR_008394) and scikit‐learn (v1.6.1; RRID:SCR_002577). All reported metrics were rounded to three decimal places.

### 4.7 Time‐Course Amino Acid Profiling in Conditioned Media

Colorectal cancer cell lines (HCT116, DLD1, SW480, and SW620) were seeded at equal densities in RPMI‐1640 medium (Welgene, LM011‐01) supplemented with 10% fetal bovine serum (HyClone, SV30207.02) and 1% penicillin–streptomycin (Gibco, 10378016). HCEC cells were initially seeded in DMEM/F12 medium (Welgene, LM002‐04) supplemented with 20% fetal bovine serum and 1% penicillin–streptomycin. The following day, the culture medium of all cell lines was replaced with RPMI‐1640 medium supplemented with 10% fetal bovine serum and 1% penicillin–streptomycin, and cells were further cultured at 37°C in a humidified incubator containing 5% CO_2_. Conditioned media were collected at 0, 12, 24, 36, 48, 60, and 72 h after media replacement. Collected media were centrifuged, and the supernatants were transferred to fresh tubes for subsequent ^19^F NMR‐based amino acid profiling as described above. Relative amino acid abundance was calculated by normalizing each amino acid signal to the total amino acid signal within each sample.

### 4.8 Statistical Analysis

The statistical methods used for amino acid profiling and model evaluation are described in the relevant Results sections and figure legends. For multiple group comparisons, a two‐sided Kruskal–Wallis test was performed. P‐values were adjusted using the Benjamini–Hochberg (BH) procedure to control the false discovery rate (FDR), with FDR < 0.05 considered significant. Differences in Kaplan–Meier survival curves were assessed using the log‐rank test, and differences in recurrence and metastasis rates were evaluated using a two‐sided Fisher's exact test. All statistical analyses were conducted in Python (v3.11.11; RRID:SCR_008394) using the SciPy package (v1.15.1; RRID:SCR_008058).

### 4.9 Code Availability

All analysis code used in this study, including data preprocessing, feature construction, and machine learning model development, is publicly available on GitHub (https://github.com/jeeylee/19F_NMR_AA_profiling). The repository contains scripts required to reproduce the main analyses and figures, including model training, evaluation, and visualization workflows. This ensures transparency and facilitates full reproducibility of the computational procedures described in this study.

## Author Contributions

J.‐Y.L. was responsible for study conception, methodology design, software development, formal analysis, data curation, and drafting of the manuscript. J.K., D.C., and D.K. performed the ^1^ ^9^F NMR experiments and contributed to manuscript revision. T.Y. supported patient cohort selection and curated clinical information (Supplementary Table S1). S.H.K. assisted in organizing clinical data and supported manuscript preparation. S.C.P. managed sample resources and performed experiments in colorectal cancer cell lines. E.J.P. provided patient samples. J.M.L. and H.K. supervised the study, oversaw overall project administration, and secured funding.

## Funding

This study was supported in part by the Samsung Research Funding & Incubation Center of Samsung Electronics (SRFC‐MA2002‐07), the National Research Foundation of Korea (RS‐2024‐00407383, RS‐2025‐25436925, RS‐2025‐02303891, RS‐2025‐15782968, and RS‐2022‐NR069827), a Faculty Research Grant (2024IF0030) from the Department of Surgery, Asan Medical Center, and grants (2025IP0041 and 2025IP0028) from the Asan Institute for Life Sciences, Asan Medical Center, Seoul, Korea.

## Conflicts of Interest

The authors declare no conflicts of interest.

## Supporting information




**Supporting File 1**: advs76044‐sup‐0001‐SuppMat.docx.

**Supporting File 2**: advs76044‐sup‐0002‐Data.zip.

## Data Availability

All data necessary to reproduce the analyses in this study are provided in Data S1, including amino acid profiling data and de‐identified clinical information of colorectal cancer patients. The dataset contains quantified concentrations of 18 amino acids, clinicopathological variables, follow‐up information, and outcome variables, including recurrence and metastasis status. All personally identifiable information has been removed.
