# Supplementary material for: Circulating Amino Acid Network Remodeling Reveals Systemic Metabolic Reprogramming Predictive of Colorectal Cancer Recurrence and Metastasis
Source: Adv Sci (Weinh). 2026 Jun 9:e76044. Online ahead of print. doi: 10.1002/advs.76044 (PMC13336814; doi:10.1002/advs.76044)
Supplement: Supplementary file 1 — Supporting File 1: advs76044‐sup‐0001‐SuppMat.docx. [file ADVS-9999-e76044-s002.docx]

Supporting Information

Circulating amino acid network remodeling reveals systemic metabolic reprogramming predictive of colorectal cancer recurrence and metastasis

Ji-Yeon Lee, Jumi Kim, Taehan Yoon, So Hyun Kwon, Su Chan Park, Dohyun Chun, Dohyeong Kim, Eun Jung Park*, Hyunwoo Kim* and Ji Min Lee*


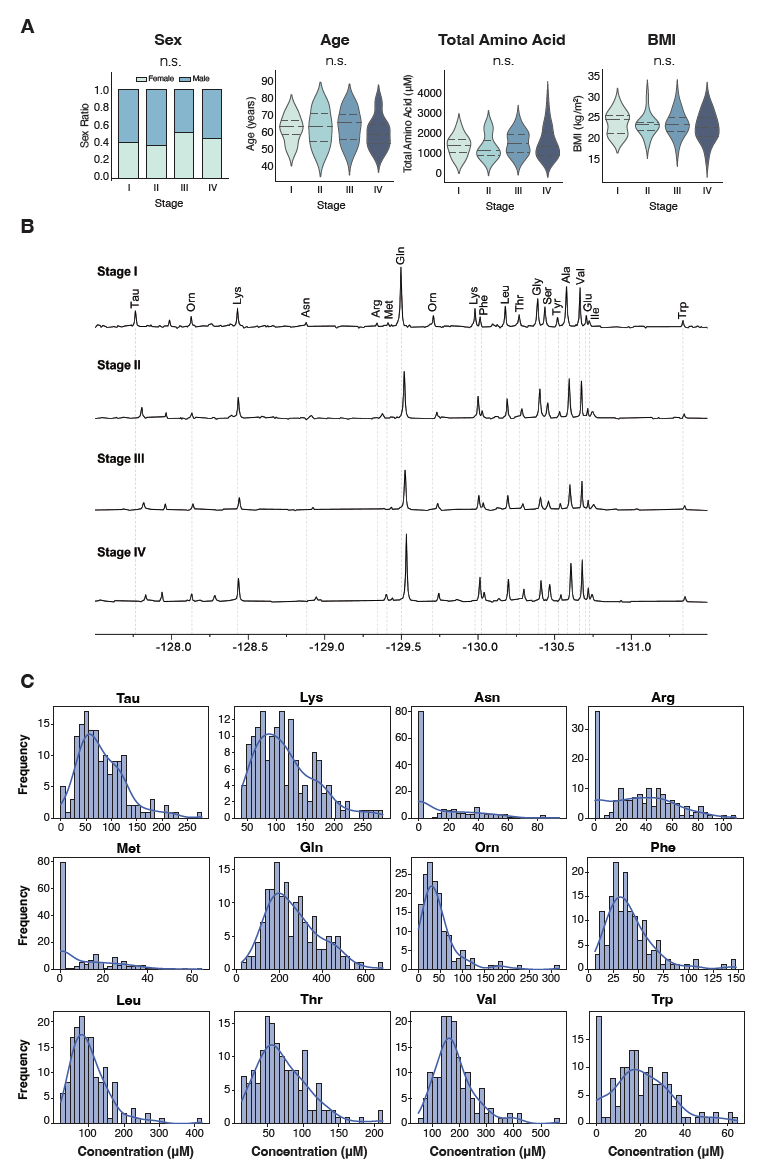


**Figure S1.**

Clinical, demographic, and NMR-based metabolic characteristics of colorectal cancer patients across TNM stages. **(A)** Clinical and demographic characteristics of the CRC patient cohort (n=152) stratified by TNM stage. From left to right, the bar graph shows sex ratio; violin plots display age, total serum amino acid concentration, and body mass index (BMI). Statistical comparisons across stages (χ² for sex; Kruskal–Wallis for continuous variables) revealed no significant differences (n.s.). **(B)** Representative ^19^F NMR spectra showing serum amino acid profiles of CRC patients across stages I–IV using fluorine-labeled analysis. **(C)** Distribution of absolute amino acid concentrations quantified using ¹⁹F NMR spectroscopy. Histograms represent the frequency of individual amino acid concentrations, overlaid with kernel density estimation (KDE) curves.


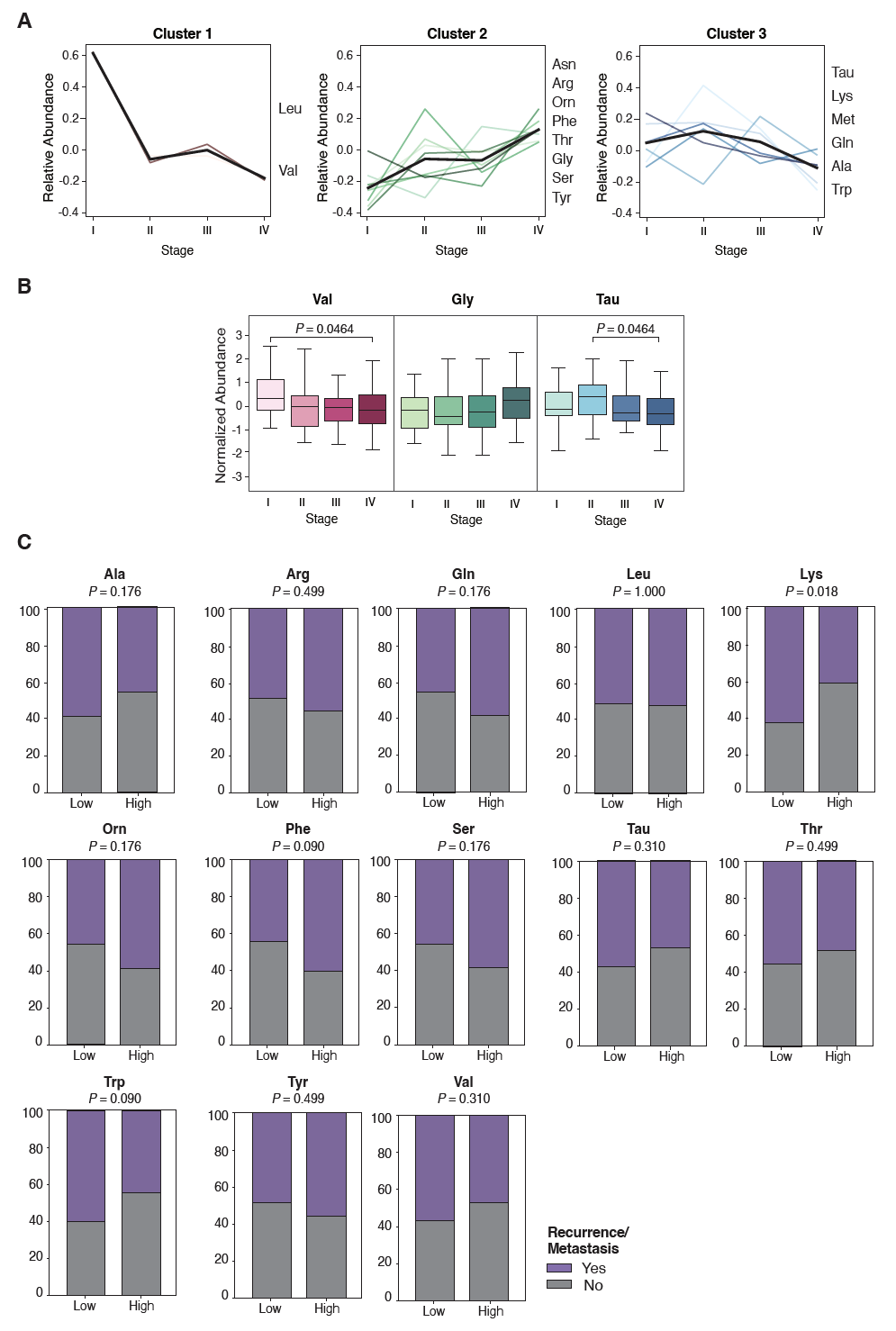


**Figure S2.**

Clustering of normalized amino acid profiles across CRC stages. **(A)** Mfuzz clustering of normalized amino acid profiles reveals distinct amino acid patterns across CRC stages. The left panel highlights amino acids with a decreasing trend (e.g., Leu, Val), the middle panel shows those with an increasing trend (e.g., Asn, Arg, Orn, Phe, Thr, Gly, Ser, Tyr), and the right panel presents another cluster of amino acids with varying trends (e.g., Tau, Lys, Met, Gln). **(B)** Box plots showing stage-specific differences in amino acid concentrations. Only amino acids with significant differences across stages are displayed. Statistical significance was assessed using the Kruskal–Wallis test followed by Dunn’s post hoc test with Benjamini–Hochberg FDR correction. **(C)** Stacked bar plots comparing recurrence or metastasis rates between low and high-abundance groups (median split) for each amino acid. Statistical significance was assessed using a two-sided Fisher’s exact test.


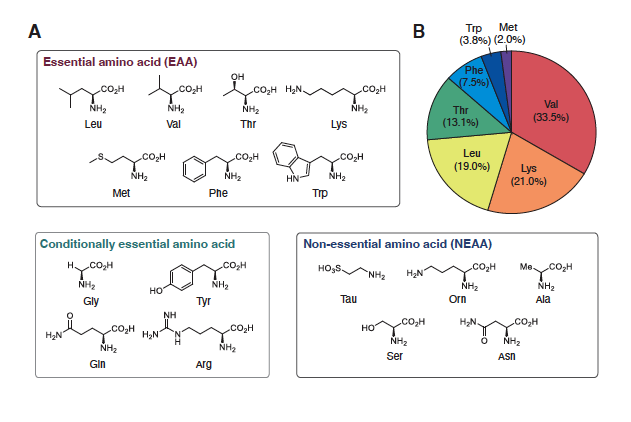


**Figure S3.**

Classification of amino acids by nutritional necessity and proportion of essential amino acids. **(A)** Amino acids detected by ¹⁹F NMR were categorized based on their nutrient necessity into EAAs (red), conditionally essential (green), and NEAAs (blue) groups. **(B)** A pie chart showing the composition of quantified EAAs in 152 CRC patients. Each section represents the relative abundance of a specific EAA.

**
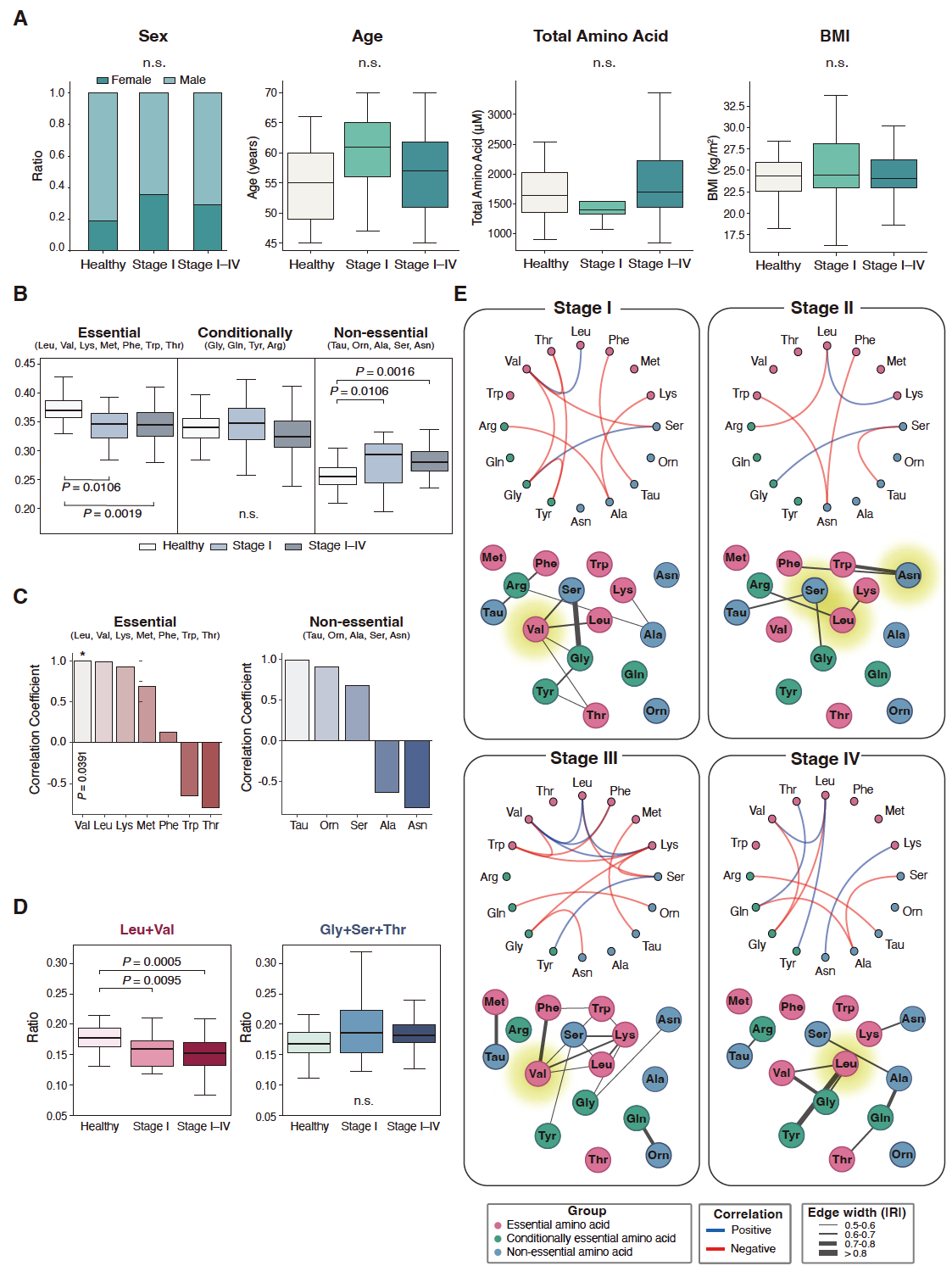
**

**Figure S4.**

External validation of amino acid class alterations and correlation network remodeling in the AMC plasma cohort. **(A)** Clinical and demographic characteristics of the cohort stratified into Healthy, Stage I, and Stage I–IV groups. From left to right, the bar plot shows sex distribution, and box plots display age, total plasma amino acid concentration, and body mass index (BMI). Statistical comparisons (χ² test for sex; Kruskal–Wallis test for continuous variables) showed no significant differences (n.s.). **(B)** Box plots showing the proportion of essential (EAAs), conditionally essential, and non-essential amino acids (NEAAs) relative to the total across Healthy, Stage I, and Stage I–IV groups. Features significant by the Kruskal–Wallis test (*P* < 0.05) were further analyzed using Dunn’s post hoc test with Benjamini–Hochberg FDR correction. **(C)** Correlation coefficient plots showing amino acids significantly associated with the EAA and NEAA ratios. Pearson correlation coefficients were calculated, with significance assessed using a two-sided Student’s t-test. **(D)** Box plots depicting group-dependent changes in representative metabolic axes across Healthy, Stage I, and Stage I–IV groups. Statistical significance was assessed using the Kruskal–Wallis test (*P* < 0.05), followed by post hoc Dunn’s test with FDR correction (Benjamini–Hochberg). **(E)** Scatter plot showing the negative correlation between BCAA levels (leucine + valine) and the combined (glycine + serine + threonine) ratio. Pearson correlation coefficients (*R*) were calculated, with significance assessed using a two-sided Student’s t-test. Shaded areas indicate 95% confidence intervals. **(F)** Network visualization of amino acid correlations across TNM stages (Stage I–IV). Nodes represent amino acids grouped as essential (pink), conditionally essential (green), and non-essential (blue), and edges indicate statistically significant correlations (|*R*| > 0.50, *P* < 0.05). Blue edges represent positive correlations, red edges represent negative correlations, and edge width corresponds to correlation strength.


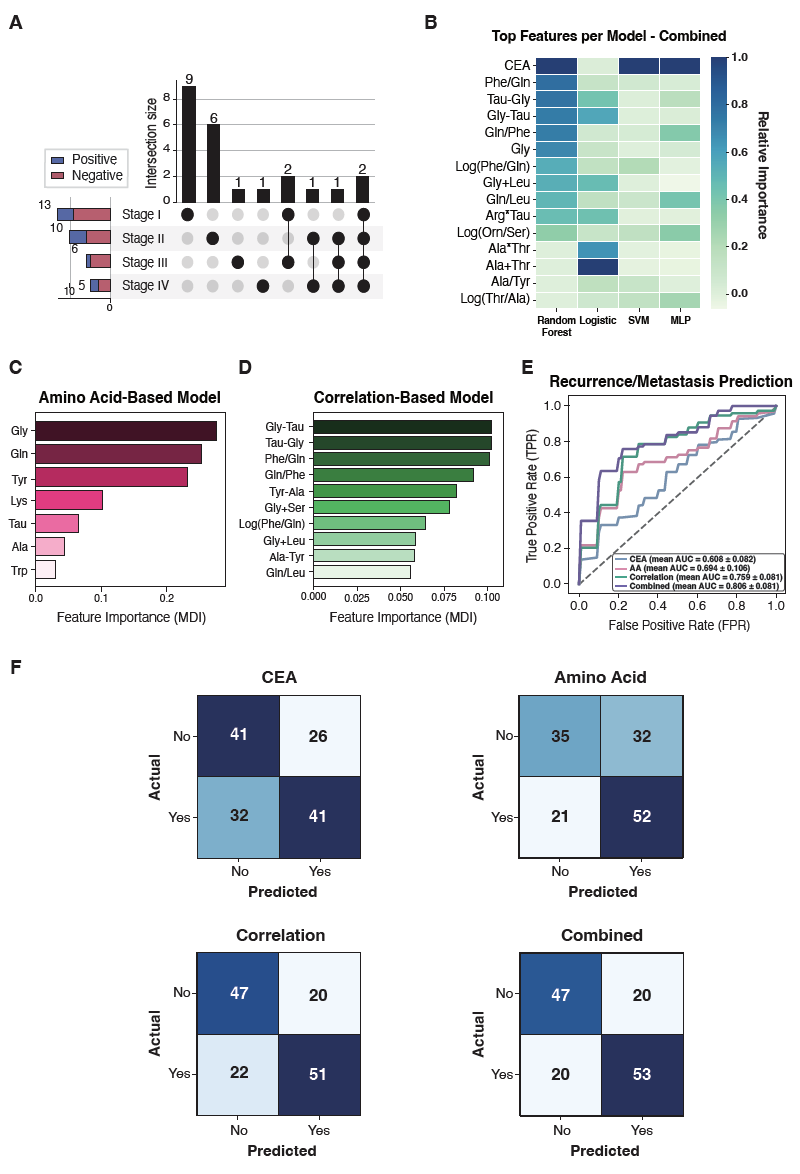


**Figure S5.**
Random forest–based prediction of recurrence or metastasis using amino acid and correlation-derived features with nested cross-validation.

**(A)** UpSet plot showing the distribution of amino acid correlations across cancer stages. Positive (blue) and negative (red) correlations are displayed, with intersection size indicating the number of shared correlations among stages. Significant correlations were defined as Pearson’s |*R*| > 0.50 and *P* < 0.05 by two-sided Student’s t-test. **(B)** Heatmap of feature importance across different machine learning models (Random Forest, Logistic Regression, SVM, and MLP) using the combined feature set. Rows represent the top features, including both individual amino acids and correlation-derived interaction features, while columns correspond to model types. Color intensity indicates normalized feature importance, highlighting features that are consistently informative across models, with correlation-based interactions showing prominent contributions. **(C)** Feature importance (MDI) of the top 7 amino acids in the amino acid–based model. **(D)** Feature importance (MDI) of the top 10 correlation features constructed from amino acid pairs in the correlation-based model. **(E)** ROC curves for recurrence or metastasis prediction using four different models: CEA, amino acid–based (AA), correlation-based (Correlation), and combined. All models were evaluated using 7-fold outer cross-validation with 4-fold inner cross-validation for hyperparameter tuning. Each curve represents the mean ROC across outer folds. The mean AUROC and 95% confidence intervals were as follows: CEA, 0.608 ± 0.082 (95% CI: 0.447–0.770); AA, 0.694 ± 0.106 (95% CI: 0.485–0.902); Correlation, 0.759 ± 0.081 (95% CI: 0.648–0.918); Combined, 0.806 ± 0.081 (95% CI: 0.648–0.964). **(F)** Confusion matrices for recurrence or metastasis prediction across four models (CEA, amino acid–based, correlation-based, and combined). Each matrix represents the aggregated classification results from the outer folds of nested cross-validation, comparing predicted versus actual outcomes (No vs Yes). The numbers indicate the count of samples in each category: true negatives (top-left), false positives (top-right), false negatives (bottom-left), and true positives (bottom-right).


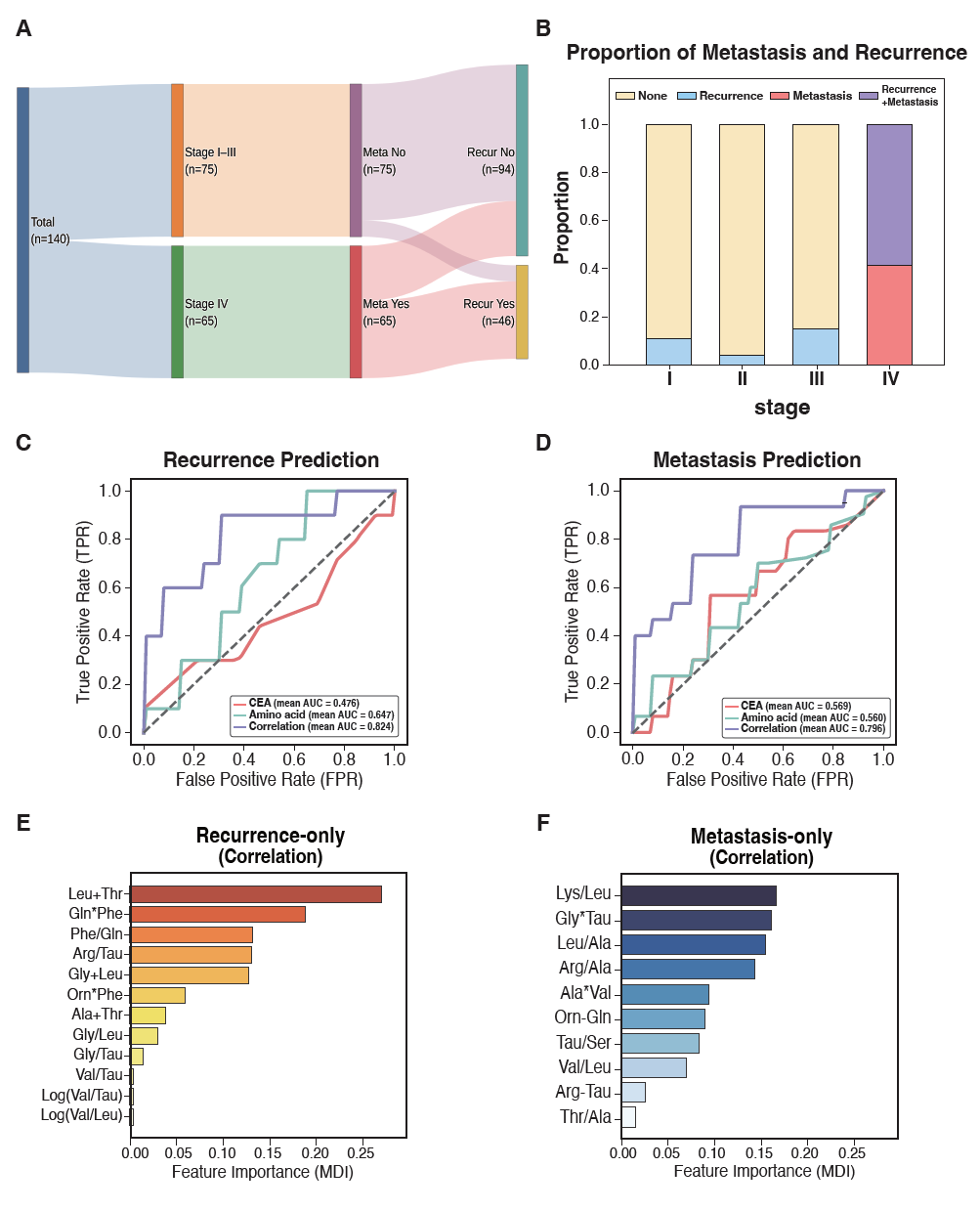


**Figure S6.**
Endpoint-specific extension of the random forest–based prediction framework for recurrence and metastasis using amino acid and correlation-derived features with nested cross-validation. **(A)** Sankey diagram illustrating the flow of patients across clinical stages, metastasis status, and recurrence outcomes. The diagram visualizes how patients transition from stage I–III versus stage IV to metastasis (Yes/No) and subsequently to recurrence (Yes/No). **(B)** Proportion of recurrence and metastasis across cancer stages. Stacked bar plots show the relative proportions of patients categorized as no event, recurrence only, metastasis only, and both recurrence and metastasis within each stage (I–IV). **(C)** Receiver operating characteristic (ROC) curves for recurrence prediction using different feature sets, including CEA, amino acid–based features (AA), and correlation-based features. Each curve represents the mean ROC across nested cross-validation folds, enabling direct comparison of predictive performance across feature types. **(D)** ROC curves for metastasis prediction using the same feature sets as in **(C)**. **(E)** Feature importance (MDI) of the top correlation-derived features in the recurrence-only prediction model. **(F)** Feature importance (MDI) of the top correlation-derived features in the metastasis-only prediction model.


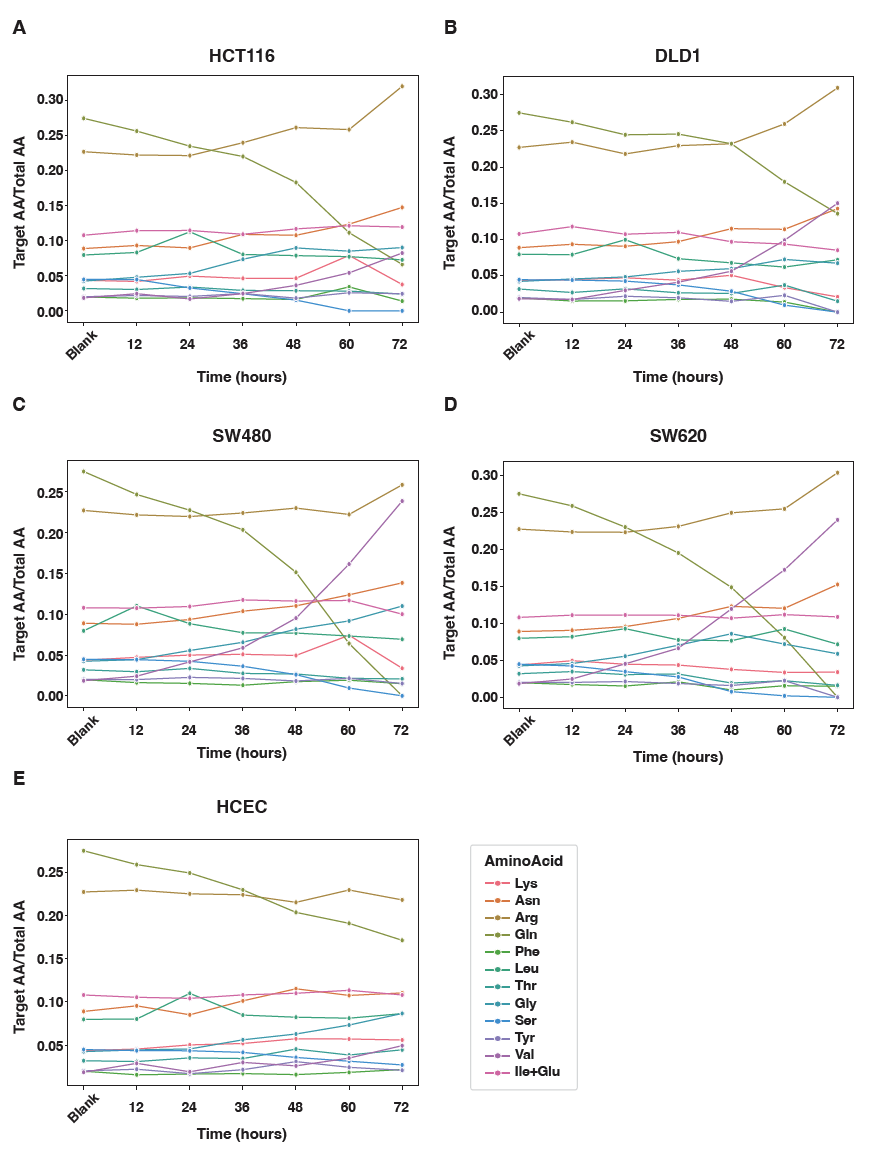


**Figure S7.**

Time-course analysis of amino acid composition in conditioned media from colorectal cancer cell lines and a normal colon epithelial cell line. **(A)** HCT116, **(B)** DLD1, **(C)** SW480, **(D)** SW620 colorectal cancer cells, and **(E)** HCEC normal colon epithelial cells were cultured under standard conditions, and conditioned media were collected at the indicated time points (0–72 h). Relative amino acid composition (Target AA/Total AA) in conditioned media was analyzed using ^19^F NMR spectroscopy. Amino acid abundance was normalized to the total amino acid signal within each sample. Data are presented as the relative proportion of each amino acid at each time point.

**Supplementary Table 1. Baseline Characteristics of the Patients**

Patients were retrospectively selected from those diagnosed with CRC and who underwent surgery at Gangnam Severance Hospital between 2015 and 2022. A total of 152 patients were included based on the following criteria: absence of synchronous malignancies, having undergone elective rather than emergency surgery, and availability of preoperative serum samples collected immediately before surgery.

| Characteristic | Patients (N=152) |
| --- | --- |
| Age — yr | |
| Mean | 61.8 ± 9.4 |
| Range | 45 - 80 |
| Sex — no. (%) | |
| Male | 85 (55.9%) |
| Female | 67 (44.1%) |
| Primary Tumor Location — no. (%) | |
| Sigmoid colon | 51 (33.6%) |
| Ascending colon | 43 (28.3%) |
| Rectosigmoid junction | 21 (13.8%) |
| Descending colon | 12 (7.9%) |
| Transverse colon | 10 (6.6%) |
| Cecum | 7 (4.6%) |
| Rectum | 5 (3.3%) |
| Hepatic flexure | 2 (1.3%) |
| Splenic flexure | 1 (0.7%) |
| Stage (pTNM) — no. (%) | |
| Stage I | 22 (14.5%) |
| Stage II | 30 (19.7%) |
| Stage III | 35 (23.0%) |
| Stage IV | 65 (42.8%) |
| Co-Existing Conditions — no. (%) | |
| Hypertension (HTN) | 48 (31.6%) |
| Diabetes Mellitus (DM) | 23 (15.1%) |
| COPD | 4 (2.6%) |
| CAOD | 3 (2.0%) |

**Supplementary Table 2. Stage-Specific Mean and Standard Deviation of Serum Amino Acid Concentrations in Colorectal Cancer Patients**

| Amino Acids | Concentration (µM) | | | | | | | |
| --- | --- | --- | --- | --- | --- | --- | --- | --- |
|  | **Stage I** | | **Stage II** | | **Stage III** | | **Stage IV** | |
|  | **Mean** | **Std** | **Mean** | **Std** | **Mean** | **Std** | **Mean** | **Std** |
| Tau | 70.82 | 31.71 | 88.96 | 45.51 | 91.92 | 57.82 | 73.56 | 45.86 |
| Lys | 108.00 | 31.74 | 107.75 | 44.06 | 123.16 | 50.38 | 116.42 | 59.30 |
| Asn | 12.68 | 18.88 | 9.96 | 18.77 | 21.81 | 23.13 | 20.07 | 23.88 |
| Arg | 28.09 | 24.33 | 32.12 | 28.84 | 35.88 | 26.17 | 36.43 | 26.32 |
| Met | 10.63 | 13.12 | 8.29 | 14.87 | 14.25 | 14.03 | 10.92 | 14.53 |
| Gln | 249.02 | 125.15 | 254.36 | 115.67 | 278.02 | 124.56 | 264.14 | 121.18 |
| Orn | 33.80 | 28.20 | 41.47 | 35.67 | 42.99 | 39.42 | 51.95 | 53.92 |
| Phe | 36.52 | 16.48 | 35.70 | 20.21 | 40.75 | 17.74 | 45.59 | 29.94 |
| Leu | 119.90 | 71.40 | 91.83 | 38.34 | 102.69 | 36.47 | 104.47 | 66.19 |
| Thr | 61.72 | 39.71 | 68.96 | 28.30 | 72.85 | 33.09 | 75.60 | 41.56 |
| Gly | 107.12 | 66.94 | 115.06 | 104.05 | 122.71 | 89.12 | 150.79 | 108.80 |
| Ser | 65.09 | 27.33 | 72.84 | 40.31 | 80.78 | 35.28 | 84.71 | 46.83 |
| Tyr | 34.75 | 17.99 | 30.36 | 17.15 | 36.09 | 17.21 | 40.26 | 27.14 |
| Ala | 193.02 | 81.64 | 192.22 | 101.50 | 210.75 | 92.15 | 213.07 | 129.39 |
| Val | 211.04 | 91.18 | 162.04 | 54.92 | 187.50 | 50.98 | 182.00 | 90.67 |
| Ile+Glu | 100.81 | 69.26 | 73.76 | 38.96 | 117.68 | 61.28 | 119.09 | 83.04 |
| Trp | 22.47 | 10.71 | 18.43 | 13.27 | 22.13 | 12.30 | 20.90 | 16.15 |

**Supplementary Table 3. Comparative Performance of Predictive Models for Recurrence or Metastasis Based on Serum Amino Acid Features**

1. CEA

| Model | Mean  AUC | Std  AUC | Mean PR AUC | Mean Accuracy | Mean Sensitivity | Mean Specificity | Mean Precision | Mean F1 |
| --- | --- | --- | --- | --- | --- | --- | --- | --- |
| Logistic | 0.723 | 0.065 | 0.770 | 0.636 | 0.416 | 0.883 | 0.817 | 0.530 |
| MLP | 0.723 | 0.065 | 0.770 | 0.586 | 0.557 | 0.640 | 0.714 | 0.549 |
| SVM | 0.396 | 0.181 | 0.521 | 0.543 | 0.957 | 0.086 | 0.541 | 0.685 |
| Random Forest | 0.608 | 0.082 | 0.663 | 0.586 | 0.565 | 0.614 | 0.622 | 0.568 |

1. AminoAcid

| Model | Mean  AUC | Std  AUC | Mean PR AUC | Mean Accuracy | Mean Sensitivity | Mean Specificity | Mean Precision | Mean F1 |
| --- | --- | --- | --- | --- | --- | --- | --- | --- |
| Logistic | 0.676 | 0.150 | 0.721 | 0.600 | 0.739 | 0.446 | 0.588 | 0.646 |
| MLP | 0.610 | 0.112 | 0.655 | 0.564 | 0.830 | 0.263 | 0.552 | 0.660 |
| SVM | 0.451 | 0.182 | 0.552 | 0.550 | 0.843 | 0.214 | 0.545 | 0.653 |
| Random Forest | 0.694 | 0.106 | 0.743 | 0.621 | 0.712 | 0.521 | 0.616 | 0.656 |

1. Correlation

| Model | Mean  AUC | Std  AUC | Mean PR AUC | Mean Accuracy | Mean Sensitivity | Mean Specificity | Mean Precision | Mean F1 |
| --- | --- | --- | --- | --- | --- | --- | --- | --- |
| Logistic | 0.683 | 0.146 | 0.734 | 0.636 | 0.768 | 0.487 | 0.625 | 0.685 |
| MLP | 0.620 | 0.151 | 0.695 | 0.514 | 0.674 | 0.341 | 0.446 | 0.521 |
| SVM | 0.600 | 0.156 | 0.638 | 0.600 | 0.810 | 0.375 | 0.588 | 0.676 |
| Random Forest | 0.759 | 0.081 | 0.771 | 0.700 | 0.701 | 0.700 | 0.725 | 0.707 |

1. Combined

| Model | Mean  AUC | Std  AUC | Mean PR AUC | Mean Accuracy | Mean Sensitivity | Mean Specificity | Mean Precision | Mean F1 |
| --- | --- | --- | --- | --- | --- | --- | --- | --- |
| Logistic | 0.660 | 0.050 | 0.699 | 0.643 | 0.629 | 0.652 | 0.695 | 0.638 |
| MLP | 0.705 | 0.116 | 0.768 | 0.621 | 0.729 | 0.513 | 0.638 | 0.668 |
| SVM | 0.685 | 0.191 | 0.766 | 0.550 | 0.935 | 0.137 | 0.544 | 0.684 |
| Random Forest | 0.806 | 0.081 | 0.830 | 0.714 | 0.727 | 0.697 | 0.737 | 0.722 |

**Supplementary Table 4. Performance Comparison of Feature Sets for Recurrence or Metastasis Using Serum Amino Acid Profile**

| Feature Set | Mean  AUC | Std  AUC | Mean PR AUC | Mean Accuracy | Mean Sensitivity | Mean Specificity | Mean Precision | Mean F1 |
| --- | --- | --- | --- | --- | --- | --- | --- | --- |
| CEA | 0.608 | 0.082 | 0.663 | 0.586 | 0.565 | 0.614 | 0.622 | 0.568 |
| AminoAcid | 0.694 | 0.106 | 0.743 | 0.621 | 0.712 | 0.521 | 0.616 | 0.656 |
| Correlation | 0.759 | 0.081 | 0.771 | 0.700 | 0.701 | 0.700 | 0.725 | 0.707 |
| Combined | 0.806 | 0.081 | 0.830 | 0.714 | 0.727 | 0.697 | 0.737 | 0.722 |

**Supplementary Table 5. Comparative Performance of Predictive Models for Recurrence and Metastasis Using Separately Defined Endpoints Based on Serum Amino Acid Features**

***-* Recurrence only**

| Model | Mean  AUC | Std  AUC | Mean PR AUC | Mean Accuracy | Mean Sensitivity | Mean Specificity | Mean Precision | Mean F1 |
| --- | --- | --- | --- | --- | --- | --- | --- | --- |
| CEA | 0.476 | 0.275 | 0.233 | 0.293 | 0.700 | 0.256 | 0.082 | 0.145 |
| AminoAcid | 0.647 | 0.190 | 0.304 | 0.720 | 0.100 | 0.788 | 0.050 | 0.067 |
| Correlation | 0.824 | 0.188 | 0.610 | 0.773 | 0.700 | 0.789 | 0.290 | 0.389 |

**- Metastasis only**

| Model | Mean  AUC | Std  AUC | Mean PR AUC | Mean Accuracy | Mean Sensitivity | Mean Specificity | Mean Precision | Mean F1 |
| --- | --- | --- | --- | --- | --- | --- | --- | --- |
| CEA | 0.569 | 0.108 | 0.276 | 0.575 | 0.567 | 0.565 | 0.207 | 0.299 |
| AminoAcid | 0.560 | 0.075 | 0.320 | 0.512 | 0.500 | 0.504 | 0.175 | 0.256 |
| Correlation | 0.796 | 0.189 | 0.615 | 0.675 | 0.600 | 0.687 | 0.277 | 0.371 |

**Data S1. (separate file)**

This file contains the integrated dataset used in this study, combining amino acid profiling and clinical information of colorectal cancer patients. It includes quantified concentrations of 18 amino acids, along with clinical variables such as sex, age, BMI, TNM stage, tumor pathology, lymphovascular invasion (LVI), perineural invasion (PNI), and preoperative chemoradiotherapy (CRT). In addition, follow-up information and outcome variables, including recurrence and metastasis status, are provided. All personally identifiable information has been excluded.
